# *Lactobacillus rhamnosus* GG-Derived Soluble Mediators Modulate Adaptive Immune Cells

**DOI:** 10.3389/fimmu.2018.01546

**Published:** 2018-07-10

**Authors:** Irene S. Ludwig, Femke Broere, Sarmauli Manurung, Tim T. Lambers, Ruurd van der Zee, Willem van Eden

**Affiliations:** ^1^Department of Infectious Diseases and Immunology, Utrecht University, Utrecht, Netherlands; ^2^Mead Johnson Pediatric Nutrition Institute, Nijmegen, Netherlands

**Keywords:** *Lactobacillus rhamnosus* GG soluble mediators, peripheral blood monocytes, dendritic cells, adaptive immunity, T cell activation

## Abstract

Probiotics and probiotic-related nutritional interventions have been described to have beneficial effects on immune homeostasis and gut health. In previous studies, *Lactobacillus rhamnosus* GG (LGG) soluble mediators (LSM) have been demonstrated to exert beneficial effects in preclinical models of allergic sensitization, bacterial infection, and intestinal barrier function. In the context of allergic diseases, differentiation of dendritic cells (DCs) and their interactions with T cell populations are crucial for driving tolerogenic responses. In this study, we set out to evaluate whether these LSM can modulate DC maturation and have an impact on prompting protective and/or tolerogenic T cell responses. Monocytes were isolated from PBMC of healthy blood donors and cultured in the presence of GM-CSF, IL-4, and LSM or unconditioned bacterial culture medium control (UCM) during 6 days to induce DC differentiation. Subsequently, these DCs were matured in the presence of TNF-α for 1 day and analyzed for their phenotype and ability to induce autologous T cell activation and differentiation to model recall antigens. After 7 days of co-culture, T cells were analyzed for activation and differentiation by flow cytometry of intracellular cytokines (IFN-γ, IL-2, IL-10, and IL-17A), activation markers (CD25), and Foxp3+ expression. LSM did not alter DC numbers or maturation status. However, these DCs did show improved capacity to induce a T cell response as shown by increased IL-2 and IFN-γ producing T cell populations upon stimulation with recall antigens. These enhanced recall responses coincided with enhanced Foxp3+ expression that was not observed when T cells were cultured in the presence of UCM-treated DCs. By contrast, the number of activated T cells (determined by CD25 expression) was only slightly increased. In conclusion, this study reveals that LSM can influence adaptive immune responses as shown by the modulation of DC functionality. These mechanisms might contribute to previous observed effects in animal models *in vivo*. Altogether, these results suggest that LSM may provide an alternative to live probiotics in case life bacteria may not be used because of health conditions, although further clinical testing is needed.

## Introduction

Dendritic cells (DCs) are sensitive to immunomodulatory effects of harmless and endogenous (microbiota) bacteria through pattern-recognition receptors such as caspase recruitment domain 15 and toll-like receptor (TLR)2 (1). DCs resulting from these interactions may present antigens such as allergens, gut microbial content (bacterial DNA, antigen, or heat shock proteins), or self-antigens in an immunomodulatory manner. For example, probiotic bacteria were shown to induce IL-10 producing regulatory T cells through dendritic cell-specific intercellular adhesion molecule-3-grabbing non-integrin-mediated DC modulation (2).

Accumulating evidence shows that in addition to the bacteria themselves, secreted components of the bacteria are capable to exert immunomodulatory effects (3, 4). In particular for *Lactobacillus rhamnosus* GG (LGG), culture supernatants were shown to protect intestinal epithelial cells from apoptosis, to promote their proliferation, and to attenuate alcohol or hypoxia-induced impaired epithelial cell resistance and permeability *in vitro* (5–7). These benefits have been linked to structural components or bioactive compounds, for example, immunomodulatory effects of the pili structure, stimulation of cell proliferation and protection from apoptosis induced by secreted proteins p40 and p75, and improved stress adaptability in the host by LGG exopolysaccharides [reviewed by Segers and Lebeer (8)]. More recently, LGG soluble factors, not necessarily linked to any specific components, have been shown to activate the type-1 immune responsiveness polarizing capacity measured in antigen-presenting cells (APCs) (9). These observations support our previous work with soluble mediators obtained from the late-exponential growth phase (LEG) of LGG on improved allergic airway inflammation in an ovalbumin-induced acute allergic airway inflammation mouse model and reduced local and systemic inflammation among neonatal rats in a model for short bowel syndrome (10, 11).

The effects of soluble mediators appear not to be limited to LGG bacteria as supernatant from other probiotic strains were shown to manifest similar immune-modulatory activities. For example, supernatant obtained from *L. reuteri* grown in tryptophan containing medium was shown to stimulate, through the aryl hydrocarbon receptor, CD4+ T cells into double-positive intraepithelial lymphocytes (DP IELs), which express both CD4 and CD8αα (12). These DP IELs are known to promote tolerance to dietary antigens.

In this study, we have analyzed the effects of LGG soluble mediators (LSM) on human DC differentiation, maturation, and activation to further explain observed immune-modulatory effects in animal models and to provide initial support for possible activity in humans. In addition, we have analyzed the effects of LSM exposed DCs on T cell populations. Altogether, we have obtained evidence that LSM are capable of inducing activated human Foxp3+ T cells *via* DC modulation in line with previous observations in mouse models.

## Materials and Methods

### Bacterial-Conditioned Medium

*Lactobacillus rhamnosus* GG soluble mediators were provided by Mead Johnson Nutrition (The Netherlands). Soluble mediators were prepared as described previously (10). In short, supernatant of a LGG culture was collected during the LEG when bacterial density reached 8 × 10^9^ CFU/mL, desalted, sterile filtered, and lyophilized. The LSM were reconstituted in milliQ water to original volume and further diluted in DC growth medium. unconditioned bacterial culture medium (UCM; media without LGG culture) underwent the same process.

### Ethical Statement

Human cells used in this study were obtained from healthy blood bank donors (Sanquin, The Netherlands). Peripheral blood from anonymous, healthy volunteers was obtained with informed, written consent in accordance with Dutch regulations. This study was approved by the Sanquin Ethical Advisory Board in accordance with the Declaration of Helsinki.

### Antibodies

Anti-human anti-CD14-VioBlue (TÜK4), CD86-FITC (FM95), CD25-APC (4E3), CD154-FITC (5C8), HLA-DR-PerCP (AC122), CD83-APC (HB15), CD11c-PE (MJ4-27G12), and IFN-γ-PE (45-15, all Miltenyi Biotech). CD40-PE-Cy™7 (5C3), IL-2-PerCP-eFluor™ 710 (MQ1-17H12), IL-10-PerCP-eFluor™ 710 (JES3-9D7), IL-4-PE (8D4-8), FoxP3-eFluor^®^ 450 (236A/E7, all eBioscience), CD4-BV510 (SK3), and IL-17A-PE (SCPL1362, BD Bioscience).

### DC Culture

CD14+ monocytes were isolated from peripheral blood monocytes (PBMCs). In short, peripheral mononuclear cells were isolated by a Ficoll gradient, and subsequently monocytes were isolated using anti-CD14 microbeads (Miltenyi Biotech). Immature DCs were generated by culturing monocytes in X-VIVO15 medium (Lonza) supplemented with penicillin (Gibco; 100 U/mL), streptomycin (Gibco; 100 µg/mL), interleukin-4 (Miltenyi Biotech; 25 ng/mL), and granulocyte-macrophage colony-stimulating factor (Miltenyi Biotech; 100 ng/mL) for 6 days. At day 3, cells were harvested and replated in 48-well plates (0.3 × 10^6^ cells/well) with fresh cytokines. During these 6 days, cells were exposed to 0.00032, 0.0016, 0.008, and 0.04% LSM or UCM.

At day 6, DCs were either directly analyzed by flow cytometry or matured with TNF-α (25 ng/mL) overnight. The phenotype of the cultured DCs was analyzed on a FACSCanto II Flow cytometer (BD Biosciences) and with FlowJo v7.6.5 (Tree Star) by addressing CD11c, CD14, CD40, CD83, CD86, and HLA-DR expression.

For co-culture studies, DCs were matured and incubated with human influenza peptide and tetanus toxoid (TT) at day 6. At day 7, supernatant was collected for IL-6 measurement, and DCs were used in DC T cell co-culture.

### IL-6 ELISA

IL-6 was measured in day 7 supernatant using an eBioscience ELISA kit according to the manufacturer’s instructions.

### DC T Cell Co-Culture

Autologous CD4+ T cells were isolated from cryo preserved PBMC by negative selection (CD4+ T cell Isolation Kit, Miltenyi Biotech). CD4+ T cells (1.2 × 10^6^) were added to the 48-well plates with maturated Ag-loaded [influenza hemagglutinin (HA) and TT] DCs and cultured for 6 days. Four hours prior to analysis, T cells were treated with PMA/ionomycin (10 ng/mL and 2 µg/mL, respectively, Sigma) and Brefeldin A (1 µg/mL, Sigma). T cells were analyzed for phenotype and intracellular cytokines by flow cytometry. For T cell analysis, single cells were gated on FSC (H) × FSC (A) scatter plot followed by a live gate based on a FSC (A) × SSC (A) scatter plot. Subsequently, different markers were compared in the CD4+ cell population.

### Statistical Analysis

To address dose dependency, significant differences were analyzed with ANOVA and a Bonferroni post-test. Data were considered significantly different with a *p* < 0.05.

## Results

**Figure 1:**
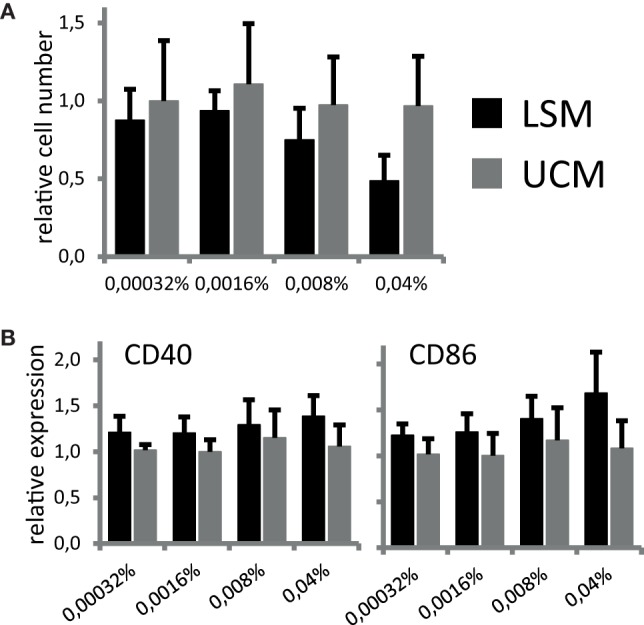
LGG soluble mediator (LSM) does not affect dendritic cells after 6 days of differentiation as described in Section “Materials and Methods” in the presence of LSM or UCM. **(A)** Relative number of CD11c+ cells after 6 days of culture. Cell counts for each incubation condition were divided by the number of cells cultured in control condition (without addition of LSM or UCM). All cells harvested at day 6 were CD11c+. **(B)** Expression of CD40 and CD86, based on mean fluorescence intensity, after 6 days of culture with LSM or UCM relative to expression on control cells. Data are presented as average of at least three independent cultures ± SEM (*p < 0.05).

To address if LSM can directly influence differentiation of monocytes into DCs, CD11c expression and CD14 expression were assessed at day 6 of culture. Cells expressing CD11c and not CD14 were considered potential DCs. No significant differences could be detected in the number of cells expressing CD11c after 6 days of culture between cells incubated in the presence of LSM compared with unconditioned bacterial culture medium (UCM). The total yield of DCs conditioned in the presence of LSM at the highest concentration was slightly lower compared with UCM (not significant) (Figure 1A).

Since both soluble and non-soluble bacterial mediators can not only influence DC differentiation but also induce maturation in APCs, for example, by stimulation of pattern-recognition receptors, we studied potential direct immunomodulatory actions of LSM on our DC culture. DC differentiated from monocytes at day 6 of culture in the presence of LSM showed a non-significant increase in CD86 and CD40 expression compared with DC differentiated in UCM (Figure 1B).

**Figure 2:**
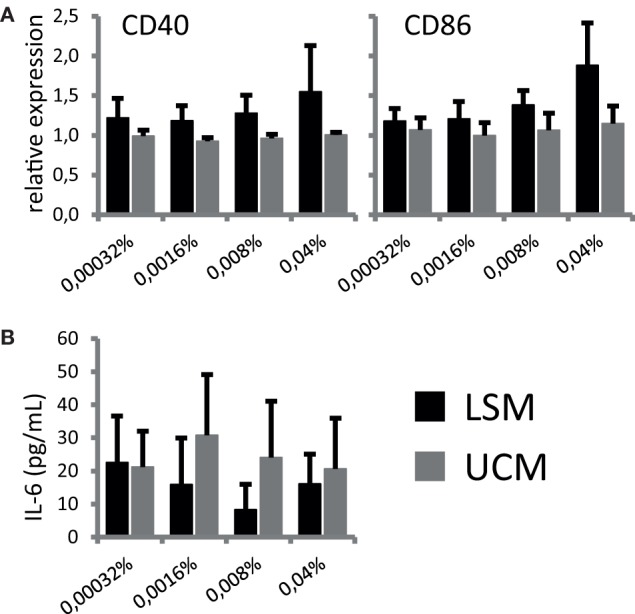
LGG soluble mediator (LSM) alters dendritic cells activation. moDC were cultured in the presence of LSM or UCM and subsequently matured by TNF-α. **(A)** Expression of CD40 and CD86, based on mean fluorescence intensity, on CD11c+ cells after overnight TNF-α stimulation relative to expression on control CD11c+ cells. **(B)** Concentration of IL-6 in culture supernatant after overnight TNF-α stimulation was measured by ELISA. Data are presented as the average of at least three independent cultures ± SEM.

To address if LSM could alter moDC under pro-inflammatory conditions, maturation of moDC in the presence of LSM was addressed. Overnight stimulation with TNF-α, to mimic pro-inflammatory conditions, induced expression of maturation markers CD40 and CD86 in both moDC cultured with LSM and UCM as expected. However, the expression of both CD40 and CD86 was marginally higher in LSM-treated DCs and maturation was increased in the presence of higher doses of LSM, this observation was only significantly different in the case of CD86 expression (Figure 2). Intriguingly enhanced maturation coincided with non-significantly altered IL-6 production as assessed in the supernatant of DC stimulated with TNF-α for 16 h (Figure 2B).

In summary, LSM does not affect DC differentiation from monocytes *in vitro*, but appears to provoke a more mature phenotype in these cells upon stimulation with TNF-α.

**Figure 3:**
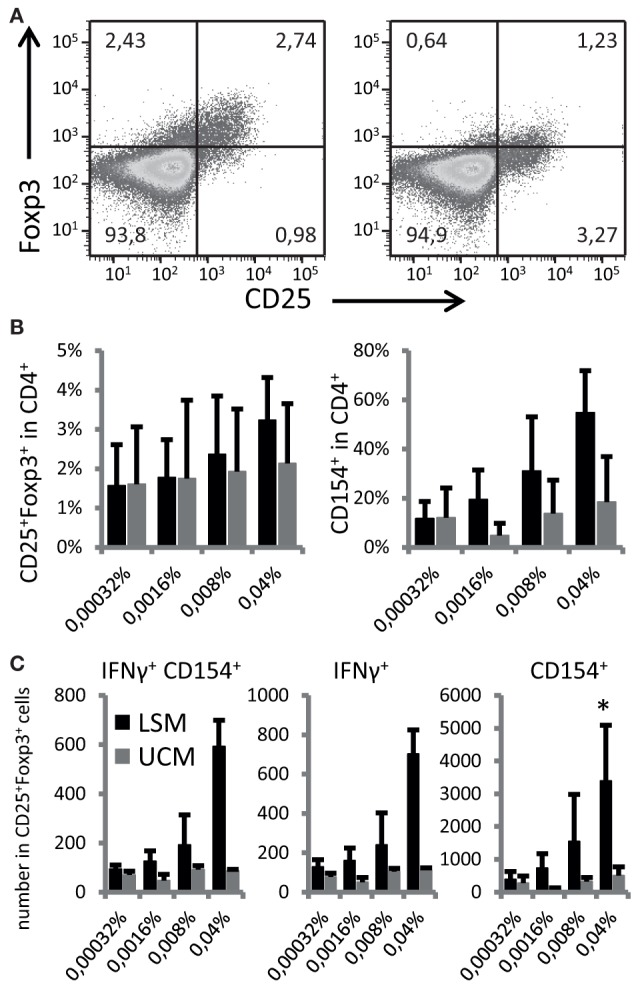
LGG soluble mediator (LSM)-modulated moDC induce enhanced Foxp3+ expression in activated T cells. **(A)** Representative plots of T cells co-cultured for 6 days with hemagglutinin (HA) and tetanus toxoid (TT) pulsed dendritic cells (DCs) that were differentiated from monocytes in the presence of LSM (left) or UCM (right). **(B)** Percentage of CD25+ Foxp3+ and CD154+ T cells after 6 days of co-culturing with HA and TT pulsed DCs that were differentiated from monocytes in the presence of LSM or UCM. **(C)** Total numbers of IFN-γ-positive and CD154-positive cells in the CD25+ Foxp3+ population in culture after stimulation. Data are average of at least three independent cultures ± SEM (*p < 0.05).

To address if the ability to enhance DC maturation also altered the antigen-presenting capacity and subsequent differentiation of responding T cells, LSM-treated moDC were cultured with autologous CD4 T cells. CD25+ Foxp3+ cells were non-significantly increased in CD4 T cells when cocultured with LSM treated DCs (Figures 3A,B). Furthermore LSM-treated moDC showed a slight non-significant increase in T cell activation capacity as shown by the upregulation of CD154 in CD4 T cells (Figure 3B).

Activation of CD25+ Foxp3+ cells was especially enhanced by moDC treated with LSM (Figure 3C). The highest concentration LSM significantly enhanced the number of Foxp3+ CD25+ cells expressing CD154 and IFN-γ (*p* < 0.05) (Figure 3C).

**Figure 4:**
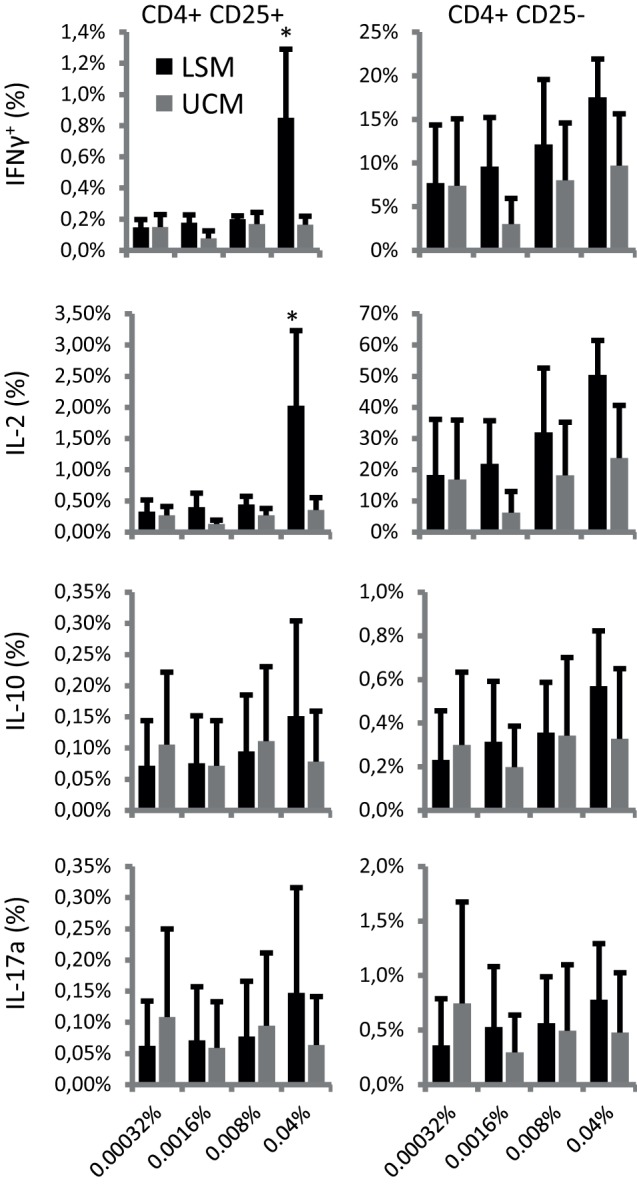
LGG soluble mediator (LSM) moDC induce enhanced intracellular cytokine expression in T cells. Intracellular IFN-γ, IL-2, IL-10, and IL-17A expression in CD4+ CD25+ (left panels) and CD4+ CD25− (right panels) T cells after incubation for 6 days with hemagglutinin and tetanus toxoid pulsed dendritic cells that were differentiated from monocytes in the presence of LSM or UCM. Data are presented as average positive cells of total CD4+ cells of at least three different donors ± SEM (*p < 0.05).

Finally, T cell differentiation as reflected by cytokine profile was studied after 6 days of stimulation with recall antigens influenza HA and TT. Intracellular cytokine expression was analyzed in CD25+ and CD25− cells. Remarkably, both CD25+ and CD25− cells showed enhanced cytokine expression when restimulated by LSM-treated moDC in the presence of recall antigens (Figure 4). LSM significantly enhanced the ability of DC to induce IFN-γ and IL-2 in CD4+ CD25+ T cells (*p* < 0.05).

## Discussion

Probiotics have been gaining substantial interest as potential immunomodulatory agents and their mode of action is increasingly being studied. To date, a range of studies demonstrate that, at least in part, some of the observed effects are mediated by probiotic-derived soluble factors. In this study, we report the immunomodulatory effects of LSM on APCs derived from healthy donors. Our results showed that LSM did not induce maturation of DC, but that these modulated DCs subsequently could induce normal T cell activation and increased activation levels of Foxp3+ T cells.

In this study, LSM did not alter DC differentiation from monocytes *in vitro*. Both during the development of the immune system and under condition of inflammation circulating monocytes leave the bloodstream and migrate into tissues where, by induction of local factors, differentiate into macrophage and DC populations. Especially, in the intestine, which will be the most prominent site for interactions with probiotics and LSM, these populations are thought to play an essential role in mucosal homeostasis and inflammation. LGG has been described to reduce TNF-α production by murine macrophage line RAW 264.7 without affecting IL-10 induction (13). By contrast, Braat et al. reported that exposure of DCs to *L. rhamnosus* during the proliferation phase, modulated DC maturation, and subsequently hampered T cell activation, as measured by decreased cytokine secretion and proliferation (14). Another group disclosed that LGG-derived soluble factors induced similar effects when compared with the viable bacteria in influencing APC activation and DC immune polarization (9). In those studies, a decrease in activation markers, cytokine secretion, and TLR expression was observed. The differences in T cell activation and DC maturation could be partly explained by our experimental set up, considering that we used soluble mediators without the whole probiotic bacteria (neither viable nor heat inactivated) and we confirmed functional relevance of LSM-modulated DCs subsequently on T cell populations isolated from the same pool of donors. Moreover, next to a trend in enhanced T cell proliferation we also observed altered T cell differentiation as shown by the cytokine profile and surface marker expression.

Although the exact bioactive components in LSM remain to be elucidated, TLR2 signaling might be involved. DCs are known to be sensitive to immunomodulatory effects of harmless bacteria through, e.g., TLR2 (1). The DCs resulting from interactions with the bacteria may present antigens such as allergens, gut microbial content (bacterial DNA, antigen, or heat shock proteins), or self-antigens in an immunomodulatory manner (1). In addition, it has been shown that LSM are a more potent TLR2 stimulus than viable LGG in an allergy mouse model (10) and immunomodulatory effects of LGG have been TLR 2 associated (6, 15).

Similar to the observations with LSM in this study, supernatant from *Bifidobacterium breve* C50 stimulated DCs and induced T cell proliferation (16). Another probiotic strain, *L. paracasei* or its supernatant reportedly stimulated DC and suppressed T cell inflammatory cytokine production (17). In an infection related design, Bermudez-Brito et al. found that *Lactobacillus rhamnosus* CNCM I-4036 supernatant was more effective in lowering pro-inflammatory cytokine secretion, including TNF-α, IL-1β, and IL-6, in *E. coli*-challenged DCs than the viable probiotics (18). Overall, these studies reveal that soluble factors generated by the respective bacteria are able to modulate the immune system, and for specific strains they may even be more effective, when compared with the viable probiotic.

*Lactobacillus rhamnosus* GG or its soluble factors have been shown to increase IL-10 production by pooled PBMC-derived monocytes/macrophages or in a DC PBMC co-culture setup (9, 19). However, we could not detect any significant increase in the production of this anti-inflammatory cytokine. A possible explanation can be a difference in preparation of the soluble mediators and in the setup of the culture experiments. However, we detected a strong Th1 response upon stimulation, as shown by the increase in IFN-γ production in CD154+ activated T cells and the IFN-γ concentration in the supernatant. A similar observation has been described previously (9, 10).

Exploration of the immunomodulatory effect of LSM in animal models showed that oral supplementation in neonatal mice reduced inflammatory mediators in the lung upon airway hyper sensitization. These data suggest that a potential immunomodulatory mechanism might be the induction of a more pronounced Th1 response, thereby preventing the induction of excessive Th2 responses resulting in immunopathology. Moreover, oral supplementation of LSM to short bowel syndrome rats recovering from intestinal resection, reduced mucosal and systemic inflammation as shown by reduced concentrations of endotoxin in the serum and lowered pro-inflammatory cytokines (IL-6 and TNF-α) in the intestinal tissue (11).

Next to animal models, LGG potentially improved influenza vaccine efficacy in healthy adults, while in children with cow’s milk allergy, LGG, when administrated together with extensively hydrolyzed casein, accelerated tolerance acquisition in cow’s milk allergic infants, and further reduced the incidence of other allergic manifestations (20–22). The immunological sequence and details by which LGG may mediate their effects remain incompletely understood. Albeit speculative results from the current study suggest that soluble mediators from LGG are able to interact with mucosal DCs and modulate the adaptive immune response that may lead to the observed clinical outcomes.

In conclusion, our data suggest that LSM contain immune-modulatory factors that promote DCs with an active T cell stimulatory capacity, which can induce T cells with both a Th1 and a regulatory phenotype. Moreover, these results suggest that LSM may provide an alternative to live probiotics in case life bacteria may not be used because of health conditions although further clinical testing is needed.

## Author Contributions

IL carried out most of the experiments. FB, SM, TL, and WE have written the paper. RZ was advisor to the study.

## Conflict of Interest Statement

The authors declare that the research was conducted in the absence of any commercial or financial relationships that could be construed as a potential conflict of interest.
